# Characterization and RNA-seq transcriptomic analysis of a *Scenedesmus obliqnus* mutant with enhanced photosynthesis efficiency and lipid productivity

**DOI:** 10.1038/s41598-021-88954-6

**Published:** 2021-06-03

**Authors:** Yimei Xi, Liang Yin, Zhan you Chi, Guanghong Luo

**Affiliations:** 1 Gansu Engineering Technology Research Center for Microalgae, Hexi University, Zhangye, 734000 People’s Republic of China; 2 School of Bioengineering, Dalian University of Technology, Dalian, 116024 People’s Republic of China

**Keywords:** Biological techniques, Biotechnology, Genetics

## Abstract

Microalgae have received significant attention as potential next-generation microbiologic cell factories for biofuels. However, the production of microalgal biofuels is not yet sufficiently cost-effective for commercial applications. To screen higher lipid-producing strains, heavy carbon ion beams are applied to induce a genetic mutant. An RNA-seq technology is used to identify the pathways and genes of importance related to photosynthesis and biofuel production. The deep elucidation of photosynthesis and the fatty acid metabolism pathway involved in lipid yield is valuable information for further optimization studies. This study provided the photosynthetic efficiency and transcriptome profiling of a unicellular microalgae, *Scenedesmus obliqnus* mutant SO120G, with enhanced lipid production induced by heavy carbon ion beams. The lipid yield (52.5 mg L^−1^) of SO120G mutant were enhanced 2.4 fold compared with that of the wild strain under the nitrogen deficient condition. In addition, the biomass and growth rate were 57% and 25% higher, respectively, in SO120G than in the wild type, likely owing to an improved maximum quantum efficiency (*F*_*v*_/*F*_*m*_) of photosynthesis. As for the major pigment compositions, the content of chlorophyll a and carotenoids was higher in SO120G than in the wild type. The transcriptome data confirmed that a total of 2077 genes with a change of at least twofold were recognized as differential expression genes (DEGs), of which 1060 genes were up-regulated and 1017 genes were down-regulated. Most of the DEGs involved in lipid biosynthesis were up-regulated with the mutant SO120G. The expression of the gene involved in the fatty acid biosynthesis and photosynthesis of SO120G was upregulated, while that related to starch metabolism decreased compared with that of the wild strain. This work demonstrated that heavy-ion irradiation is an promising strategy for quality improvement. In addition, the mutant SO120G was shown to be a potential algal strain for enhanced lipid production. Transcriptome sequencing and annotation of the mutant suggested the possible genes responsible for lipid biosynthesis and photosynthesis, and identified the putative target genes for future genetic manipulation and biotechnological applications.

## Introduction

Microalgae have been explored and have received considerable attention in recent years as potential next-generation cell factories for biofuels due to their higher photosynthetic efficiency, rapid growth rate, and a greater lipid production^1,2^. Over the past few years, several oleaginous algal species have been isolated and characterized. However, naturally occurring microalgae produce much lower amounts of lipids than the theoretical maximum, which greatly limit the commercial applications. In fact, it has also been determined that it is less practical to screen natural algal strains with higher biomass and lipid productivity. Therefore, it is necessary to explore and utilize other powerful tools to screen high lipid-yielding algal strains.

Many efforts have been made to improve screening efficiency for the purpose of obtaining industrial strains with an excellent lipid productivity. These methods include physical or chemical mutagenesis and genetic engineering. In previous research, ultraviolet (UV) or γ and X-rays exposure has been applied to isolate mutants that can provide quality improvement^2–5^. Among these methods, a heavy-ion beam is well known for its higher relative biological effectiveness (RBE) and linear energy transfer (LET)^6–8^, which can cause large deletions/insertions, translocations, or rearrangements in the genome to obtain microalgae mutants with broad spectrums and high frequencies^9,10^. Previous studies found that the biomass of *Nannochloropsis* increased by 19%, and the lipid productivity of *Desmodesmus* sp. increased by 20.6% after heavy ion irradiation^10,11^. The maximum quantum efficiency (*F*_*v*_/*F*_*m*_) of *Desmodesmus* sp. and *Nannochloropsis* has also been found to be higher than the wild strains under high light^10,11^. Therefore, all of these research results have implied that heavy ion irradiation may simultaneously increase biomass and lipid productivity in microalgae with improved photosynthesis activity. However, the mechanism of heavy-ion irradiation for microalgae has not yet been investigated, which includes the influence on microalgae lipid metabolism. In particular, the functions of key genes and enzymes involved in lipid synthesis and accumulation in these microalgal are not well understood. Moreover, the transcription factors involved in the regulation of lipid accumulation have remained unclear. Currently, genetic manipulation is an effective strategy for the enhancement of lipid overproduction by integrating and overexpressing one or more key exogenous genes related to lipid biosynthesis in microalgal strains. To perform gene manipulation in microalgal strains, their genome information is necessary. To solve the problem, RNA-seq and transcriptome analysis has been performed on mutants, and the key enzymes and key genes of various metabolic pathways have been analyzed. The RNA-seq information will help elucidate the key expression genes and provide basic data support for the mechanism explanations and microalgal synthetic biology during the later period.

*Scenedesmus obliqnus* is a ubiquitously occurring unicellular green microalgae that exhibits rapid growth and reproduction, while possessing a high lipid content under stress conditions^12^. In this study, a *S. obliqnus* mutant, SO120G, with enhanced lipid production is generated using heavy-ion irradiation. Then the desirable biomass and lipid productivity are observed under different nitrogen cultivation conditions. Photosynthetic profiling is also characterized and analyzed to elucidate the response of *S. obliqnus* to heavy-ion irradiation. Moreover, an RNA-seq and transcriptome analysis of the *S. obliqnus* mutant, SO120G, and the wild type is conducted to elucidate the differentially expressed key genes (DEGs) involved in the lipid biosynthesis pathway.

## Materials and methods

### Cultivation of microalgal samples

The *S. obliqnus* FACHB-416 strain was obtained from the Freshwater Algae Culture Collection of Hydrobiology, Chinese Academy of Sciences, China. The wild type (WT) was grown in a BG-11 culture medium under continuous illumination at a low light intensity of 50–100 µmol photons·m^−2^ s^−1^. Culture mixing was achieved though aeration using compressed air containing 2% CO_2_. All of the strains were cultured in three replicates at room temperature (25 °C).

### Heavy-ion irradiation mutagenesis

The *S. obliqnus* strain was cultivated in a BG-11 medium in a 100 mL Erlenmeyer flask under a low light illumination of 50–100 µmol photons· m^−2^ s^−1^ at room temperature for three days. Algal cells during the exponential growth phase were collected by centrifugation (3000*g*, 3 min) and washed with sterile water, and then re-suspended with fresh BG-11 medium. The cell concentration was adjusted to 1 × 10^6^ cell mL^−1^, and exposed to a ^12^C^6+^ ion beam provided by the Heavy Ion Research Facility at Lanzhou (HIRFL), Institute of Modern Physics of Chinese Academy of Sciences (CAS). The carbon ion energy was 80 meV/µ. The average LET value (the energy transferred per unit length, keV µm^−1^) was 31 keV µm^−1^. Irradiation treatments were conducted at dosages of 60, 90, and 120 Gy, referenced from the previous reports^13^, and calculated from particle fluencies and the LET. There were at least three algal samples for each dose treatment.

### Mutant isolation

After irradiation, the algal cells were plated on BG-11 agar plates in triplicate and cultured at 25 °C under low-light conditions (50 µmol photons·m^−2^ s^−1^) until the algal colonies appeared on the plates. The colonies derived from the irradiated cells were selected and transferred to BG-11 agar plates several times to obtain purified monoclonal strains, which were regarded as putative mutants and constituted the mutant library.

### Screening of lipid and photosynthesis mutants

The putative mutants and the WT were inoculated into 24-well microplates where each well contained 2.5 mL of BG-11 culture medium, and they were cultured under low-light conditions (~ 100 µmol photons· m^−2^ s^−1^) at 25 ± 2 °C for six days. On the sixth day, the OD_680nm_ of the cultures were measured using a spectrophotometer (Jasco V-530, JASCO Corporation, Japan). The 24-well microplates were subjected to a high-light intensity of 300 µmol photons· m^−2^ s^−1^ for six more days. The amount of initial inoculation with the monoclonal strain was 9% by volume. Afterwards, the 15 mL glass tubes were subjected to a high-light intensity of 300 µmol photons· m^−2^ s^−1^ for seven more days. Then the algal cells were used for the lipid content and *F*_*v*_/*F*_*m*_ screening. Three replicates of each mutant were analyzed. After large-scale screening, one mutant, named SO120G, was obtained, which had a high lipid content and photosynthesis efficiency.

### Cultivation of the microalgal mutants

After four days, the seed culture was collected using centrifugation (3000*g*, 3 min) and transferred into a 500 mL nitrogen-limited BG-11 medium (4.25 mM NaNO_3_) in column photobioreactors (100 cm high, diameter 5 cm, 500 mL culture volume). The initial OD_680nm_ of the algal culture was approximately 0.2. The wild type and mutant cultures were grown under continuous illumination of 100 µmol photons· m^−2^ s^−1^ with aeration containing 2% CO_2_ (which was referred to as N–) for 2 weeks. All of the strains were cultured in three replicates at room temperature (25 °C).

### Analytical methods

#### Growth analyses

The cell density was determined spectrophotometrically using a UV/VIS spectrophotometer (Jasco V-530, JASCO Corporation) at 680 nm. The microalgal dry weight (DW) was determined according to the method described by Cao et al.^14^. Briefly, using pre-weighed Whatman GF/C filters, a 5–10 mL culture broth was filtered and washed three times using 2 mL of 0.5 M ammonium bicarbonate and then dried < 60 °C for over 16 h until the weight was constant. The dry weight (DW) of the microalgae cells was calculated according to the difference between the final and initial filter weights and volume of the filtered sample.

The microalgal growth rate (*µ*_*i*_, d^−1^) was calculated using Eq. (1):

1 $$\mu_{i} = \frac{{LnDW_{i} - LnDW_{i - 1} }}{{t_{i} - t_{i - 1} }}.$$

The biomass productivity (*P*_*i*_, g L^−1^ d^−1^) was calculated as follows:

2 $$P_{i} = \frac{{DW_{i } - DW_{i - 1} }}{{t_{i} - t_{i - 1} }},$$

where *DW*_*i*_ and $DW_{i - 1}$ (g L^−1^) are the biomass concentration measured at time *t*_*i*_ and *t*_*i–*1_, respectively; and *t*_*i*_ and *t*_*i–*1_ are days i and I − 1, when the culture broth was sampled.

#### Measurement of the lipid, protein, and starch content

Approximately 5 mg of dry biomass (for biomass fatty acid analysis) was added to 98%: 2% (v/v) methanol: H_2_SO_4_ and incubated for 1 h at 70 °C^15^. After methylation, deionized water and hexane were added to extract the fatty acid methyl esters (FAMEs). C17:0-TAG was added as the internal standard for quantification. Separation and identification of the FAMEs were performed by gas chromatography flame ionization detection (GC-FID, Agilent 6890) with a DB-23 capillary column (30 mm × 0.32 mm × 0.25 µm). The injector temperature was 270 °C, with a split ratio of 50:1. The program for the column temperature began at 130 °C for 1 min, followed by an increase to 170 °C at a rate of 10 °C min^-1^, and then another increase at a rate of 2.8 °C min^-1^ to 215 °C, where it was maintained for 1 min. The detection temperature was 300 °C. The FAMEs were identified using the retention time and mass spectral matching*.*

The protein content was determined using the Markwell method, a modified Lowry method^16^. Briefly, lyophilized algae powder (4–5 mg) was added to 1 mL of a 0.5 mol L^−1^ NaOH solution. The prepared biomass solution was then hydrolyzed in 80 °C water bath for 10 min, and then 1 mL of distilled water was added. To obtain the protein extract, the biomass lysis solution was centrifuged at 12,000 rpm for 3 min. The above procedure was repeated twice, and the last extract was combined in a total volume of 5 mL. The protein content was determined using the Coomassie Brilliant Blue (CBB) method with bovine serum albumin (BSA) as the standard.

The starch content was measured according to the method described by Zheng et al.^17^. Briefly, the algal pellet was re-suspended in 0.1 M pH 4.4 acetate buffer and autoclaved at 110 °C for 15 min to solubilize the starch. Then, 1.5 units of amyloglucosidase (Sigma-Aldrich, St. Louis, Mo, USA) were added, and the solution was heated at 55 °C for 1 h. The glucose was determined by the sulfuric acid-anthrone method. The blank assay for starch determination was conducted by adding equal amounts of reagents to a microalgae-free sample. The starch content (% DW) was calibrated by subtracting the glucose content from a blank assay and multiplying by 0.9.

#### Pigment extraction and determination

To determine the contents of the pigments, including chlorophyll (Chl a and Chl b) and the total carotenoids, 10 mg of dried biomass was extracted with 1 mL of 90% (v/v) acetone, vortexed for 20 s, and then centrifuged at 10,000 g for 2 min. The above pigment extraction procedure was repeated several times until the algae was colorless. The absorbances at 645 nm, 662 nm, and 470 nm of the extraction solution were measured using a UV/VIS spectrophotometer (Jasco V-530, JASCO Corporation). Chl a, Chl b, and the total carotenoid contents were calculated using the equations below:

3 $${\text{Chla}}\left( {{\text{mg}} \cdot {\text{L}}^{{ - {1}}} } \right) \, = { 11}.{75 }\left( {{\text{A}}_{{{662}}} } \right) - {2}.{35 }\left( {{\text{A}}_{{{645}}} } \right)$$

4 $${\text{Chlb}}\left( {{\text{mg}} \cdot {\text{L}}^{{ - {1}}} } \right) \, = { 18}.{61 }\left( {{\text{A}}_{{{645}}} } \right) - {3}.{96}\left( {{\text{A}}_{{{662}}} } \right)$$

5 $${\text{Total}}\;{\text{carotenoids }}\left( {{\text{mg}} \cdot {\text{L}}^{{ - {1}}} } \right) = \frac{{1000{\text{A}}_{470} - 2.270{\text{ Chl a}} - 81.4{\text{ Chl b}}}}{198}$$

6 $$\begin{gathered} {\text{Pigment content }}\left( \% \right) = \hfill \\ \frac{{{\text{Pigment concentration }}\left( {{\text{mg}} \cdot {\text{L}}^{ - 1} } \right) \times {\text{Volumn }}\left( {5{\text{ mL}}} \right)}}{{{\text{M }}\left( {{\text{mg}}} \right)}} \times 0.001 \times 100{\text{\% }} \hfill \\ \end{gathered}$$

#### Chlorophyll fluorescence measurement

The PSII activity of algal cells was measured using a chlorophyll fluorometer (Water-PAM Heinz Walz GmbH, Effeltrich, Germany). First, a dark adaption of 10 min was performed before applying a saturating pulse (0.6 s, 1400 µmol m^−2^ s^−1^) to measure the maximal PSII quantum yield (*F*_*v*_/*F*_*m*_), quantum yield of PSII (ФPSII), nonphotochemical quenching (NPQ), and a relative photosynthetic electron transport rate (rETR). The *F*_*v*_/*F*_*m*_ NPQ, and rETR were determined according to the methods described by Yao et al.^18^.

### Transcriptome analysis

#### RNA extraction and sequencing

The WT *S. obliqnus* FACHB-416 and mutant *S. obliqnus* SO120G were chosen for transcriptome analysis. The total RNA was extracted from the SOWT (repeated samples, SOWT-1, SOWT-2, and SOWT-3) and the mutant SO120G group (repeated samples, SO120G-1, SO120G-2, and SO120G-3), considering the cells were active in the early stage with a lag between transcription expression. Following a previous method^19^, the total RNA in the *S. obliqnus* cells was extracted using a Total RNA Extraction System (Takara, Japan), and the mRNA was purified and fragmented to approximately 200 nt. These fragments were used as templates to synthesize the cDNA. After purification, their sequencing was performed by the Novogene Bioinformatics Technology Co. (Beijing, China).

#### Annotation of the transcriptome

The transcriptome analysis was performed by the Novogene Bioinformatics Technology Co. (Beijing, China). Clean reads obtained from editing raw reads were mapped onto unigene sequences using Bowtie2-2.2.3. The method of the expected number of fragments per kilobase of transcript sequence per millions base pairs sequenced (FPKM), as outlined by Zuo et al.^20^ was performed to quantify the gene expression levels. The DESeq R package was used to analyze the differential expression genes (DEGs) in the *S. obliqnus* cells between the SOWT and the SO120G with significantly differential expressions at a *p* value < 0.05 and fold change ≥ 2. The KEGG pathways for the DEGs were annotated using the KEGG automatic annotation server.

### Statistical analysis

The one-way ANOVA^14^ was performed in Excel (version 2013, Microsoft) to analyze the growth (OD and dry weight) and fatty acid content, carotenoids content, protein content, and starch content of the microalgae WT and SO120G.

## Results and discussion

### *S. obliqnus* mutant strain screening

*S. obliqnus* cells during the exponential growth phase were treated using a ^12^C^6+^-ion beam to induce random genomic mutations, and the colonies that appeared on the BG11 agar plates were preliminarily considered as putative mutants. The morphological characteristics (e.g., colony appearance, cell shape, and size) of the putative mutants were indistinguishable from the WT cells under light microscopy. The colonies were then transferred to 24-well microplates for amplification. The photosynthetic characteristic parameters (*F*_*v*_/*F*_*m*_ and the quantum efficiency of the PSII) of the putative mutants were further characterized using the chlorophyll fluorescence technique^10^. Previously, chlorophyll fluorescence has been used as a sensitive, quantitative parameter to analyze the photosynthetic characteristics of cultivars^21,22^, as well as to characterize microalgal mutants. Under favorable conditions, the *F*_*v*_/*F*_*m*_ value of the *S. obliqnus* WT cells was approximately 0.7. The *F*_*v*_/*F*_*m*_ values of the colonies with a significant difference from the WT were identified as possible photosynthesis efficiency mutants (PEMs). The frequencies of the PEMs induced at dosages of 60, 90, and 120 Gy of the ^12^C^6+^ beam were 10.5%, 18.8%, and 26.3%, respectively. Therefore, the dose of 120 Gy represented an optimal mutation frequency. Previous studies have indicated that the mutation rate of terrestrial plant materials, such as seeds, leaves and other organs ranged from 8.4% to 17.8%^8,23,24^ after irradiation by heavy-ion beams, which was higher than the traditional mutagenesis induced by X-ray, γ-ray, or EMS (Electro Magnetic Susceptibility)^8^. In this study, the mutation rate of *S. obliqnus* induced by a heavy-ion beam reached up to 26.3%, and this was considerably higher than those of plant materials. In addition, the mutation rate of *S. obliqnus* showed high similarity with that of *Desmodesmus *sp. from a previous report^10^. These results implied that heavy-ion irradiation could be an effective tool for the mutagenesis of microalgae.

**Figure 1:**
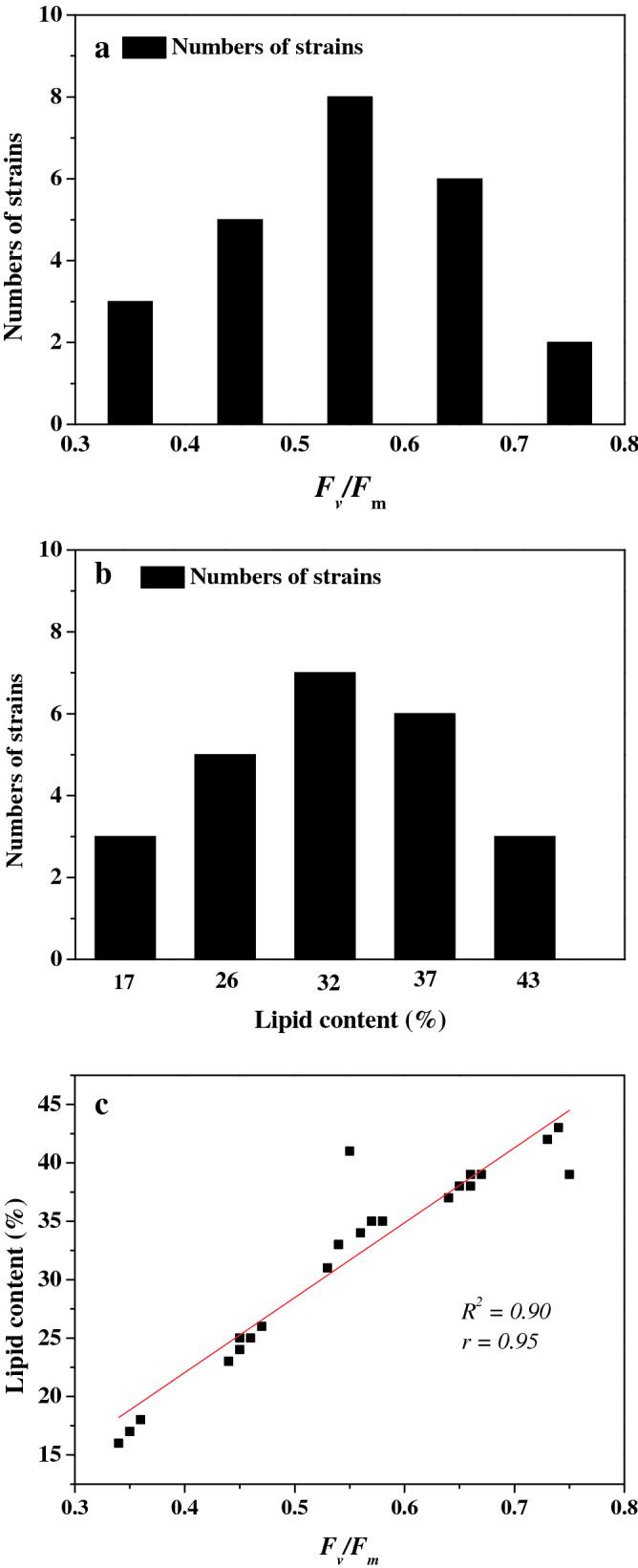
Distribution histogram of the SO120G mutant phenotypes. (**a**) Distribution histogram of the photosynthesis efficiency mutants (PEMs); (**b**) distribution histogram of the lipid-over-production mutants (LOMs); and (**c**) the relationship between the lipid contents and *F*_*v*_/*F*_*m*_ under stress conditions in the PEMs and LOMs of SO120G.

During the first-round of screening, several PEMs were obtained, and a wide range of the phenotypic distribution of these mutants were observed, which approximately agreed with the normal distribution (Fig. 1a). During the following screening procedures, the lipid contents and photosynthetic efficiency of the mutants were further assayed. The lipid content distribution of these PEMs is shown in Fig. 1b, and the contents of most of the PEMs significantly increased over that of the WT (18.1%) under stress conditions. A positive correlation (R^2^ = 0.90) existed between the lipid content and high PSII efficiency (*F*_*v*_/*F*_*m*_) under stress conditions (Fig. 1c). Here, the lipid content and high PSII efficiency (*F*_*v*_/*F*_*m*_) were evaluated together to analyze the Spearman’s rank correlation coefficient. The maximal Spearman’s rank correlation coefficient (0.95, *p* $<$ 0.01) was observed by plotting the lipid concentration against the corresponding *F*_*v*_/*F*_*m*_ (Fig. 1c), generally, if the Spearman’s rank correlation coefficient is more than 0.8, it is assumed that there is a strong correlation between two events^19^. Similar phenomena were also observed in naturally occurring microalgae strains^10^, and this indicated that the substantial accumulation of lipids in the photoautotroph was ultimately derived from increased photosynthesis. Afterwards, only those mutants with at least a 10% increase in photosynthetic efficiency and lipid content over that of the WT were selected for the next round of screening. After three to four rounds of the screening process, a mutant named SO120G with a twofold increase in photosynthetic efficiency and lipid content over that of the WT was obtained.

### Biomass and total lipid contents of the WT and mutant SO120G

**Figure 2:**
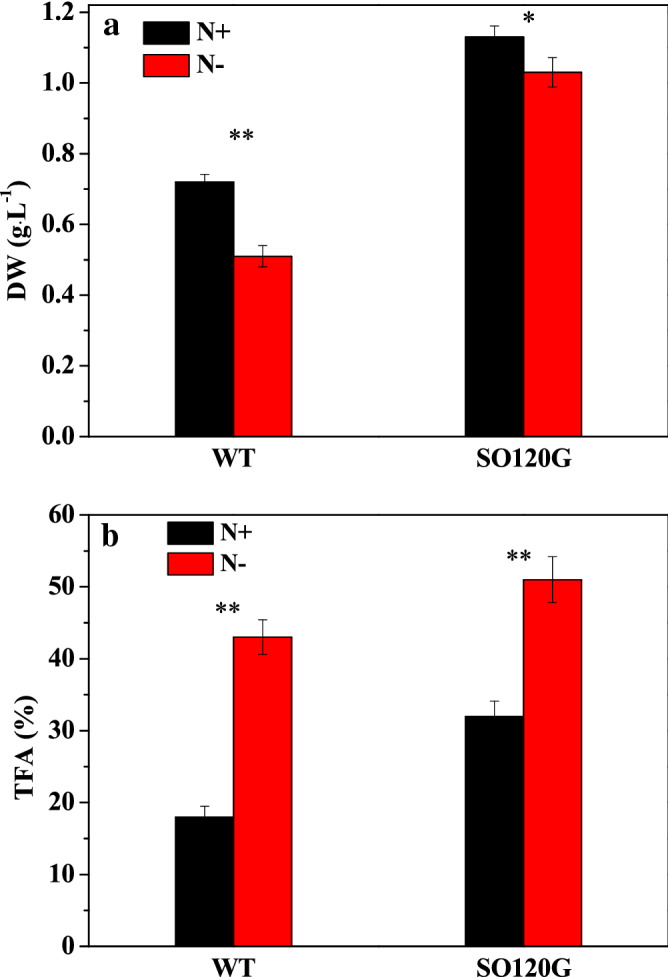
Biomass and total lipid contents of the WT and mutant SO120G under the N + and N- culture conditions. (**a**) Biomass of the WT and SO120G and (**b**) the TFA content of the WT and SO120G, *represents a significant effect (*p* < 0.05), and **represents a very significant effect (*p* < 0.01).

The *S. obliqnus* colonies were cultivated in a BG-11 medium under different nitrogen conditions and exposed to light illumination, and they grew rapidly for four to five days and reached a stationary phase thereafter (Fig. 2a). Under nitrogen sufficient conditions, the maximum dry weight (DW) concentrations of the WT and SO120G were 0.72 and 1.13 g L^−1^ (Fig. 2a), respectively, and the specific growth rates of the WT and mutant SO120G were 0.28 and 0.35 d^-1^, respectively, in seven days, which was an increase of approximately 57% and 25% over that of the WT for biomass and growth rate, respectively. Under nitrogen limitation conditions, the mutant SO120G showed a similar growth profile as the wild type (WT), and the biomass accumulations of the WT and SO120G reached up to 0.51 g L^−1^ and 1.03 g L^−1^, respectively (Table 1). In addition, a significant difference in the lipid content was observed between the WT and mutant SO120G. It is interesting to note that the SO120G produced much more total fatty acids (TFA) than the WT whenever cultivated under the nitrogen deficient (N-) or sufficient (N+) conditions (Fig. 2b). In particular, when nitrogen was deficient in the culture, after being cultivated seven days, the lipid yield of the mutant SO120G (52.5 mg L^−1^) significantly enhanced 2.4 times that of the WT (21.9 mg L^−1^). The total TFA content of the mutant SO120G was 32%, 51%, which was and 78% and 19% higher than that produced by the WT (18%, 43%) under N+ and N− culture conditions, respectively.

**Table 1:** The major biomass compositions of *S. obliqnus*.

Strains	DW (g L^−1^)	Lipid productivity (mg·L^-1^)	Protein (%DW)	Starch (%DW)
WT (N+)	0.72** (± 0.02)	18.4** (± 0.98)	42 (± 1.20)	7.3 (± 0.61)
WT (N−)	0.51** (± 0.03)	32.8** (± 0.87)	34 (± 1.030)	16.4* (± 0.18)
SO120G (N+)	1.13** (± 0.03)	37.1** (± 1.03)	36 (± 1.12)	7.2 (± 1.05)
SO120G (N−)	1.03** (± 0.04)	64.8** (± 1.30)	30 (± 0.90)	12.5*(± 1.12)

Values are means (± SD) of n = 3 cultivations per treatment, *represents a significant effect (*p* < 0.05) and **represents a very significant effect (*p* < 0.01).

In addition, Table 1 shows the fatty acid profiles of lipids in the WT and SSO120G after N+ and N− cultivation. C16–C18 fatty acids (16:0, 16:3, 18:0, 18:1, 18:3, 18:3), suitable for biodiesel production^25^, were all higher than 80%, and 18:3 was the most prevalent constituent in the WT samples, followed by 16:0 and 18:1. In addition, 18:3 exhibited no statistically significant change under both N+ and N− conditions, while 16:3 and 18:1 increased. 16:0 decreased significant during N− cultivation than the WT did under N+ cultivation. The ANOVA showed that 18:1 was the most prevalent constituent, followed by 16:0 and 16:3 in the SO120G samples, and 16:3,18:3 increased during N- cultivation than they did during N+ cultivation, while 18:1 showed no statistically significant change under both the N+ and N− conditions in SO120G. The above results show that the significant effect of the N- condition on fatty acid profiles that were also found in some previous studies^25,26^.

The previous study showed that inhibition of starch synthesis could result in a tenfold increase of TAG (triacylglycerol) in a starchless mutant of *C. reinhardtii*^27^. The TFA content of the mutant SO120G was more abundant than that of the WT. To determine whether excess accumulated TFA in SO120G might be partially at the expense of starch, the starch contents of SO120G and WT were analyzed quantitatively. After being grown under N+ conditions for seven days, the WT and SO120G accumulated a basal amount of starch (approximately 7% of cell dry weight), and then, when they were transferred to N- conditions, the starch contents of the WT and SO120G increased by 16.4% and 12.5%, respectively (Table 2). Considering that the starch synthesis pathway in the SO120G was not blocked because the mutant SO120G could synthesize an equal amount of starch as the WT, it was likely that the starch within the algal cells was partially converted to lipids. A similar conversion of the proteins in the WT and SO120G was observed, and the protein contents of the WT and SO120G cultivated under the N-conditions decreased by 34% and 30%, respectively.

**Table 2:** The major pigment compositions of *S. obliqnus*.

Strains	Chla (%)	Carotenoids (%)
WT (N+)	3.95** (± 0.08)	1.25** (± 0.05)
WT (N−)	1.83* (± 0.05)	2.05** (± 0.03)
SO120G (N+)	5.25** (± 0.07)	1.85** (± 0.08)
SO120G (N−)	2.15* (± 0.02)	2.85** (± 0.02)

Values are means (± SD) of n = 3 cultivations per treatment, *represents a significant effect (*p* < 0.05), and ** represents a very significant effect (*p* < 0.01).

### Characterization of the photosynthesis activity of the WT and SO120G

**Figure 3:**
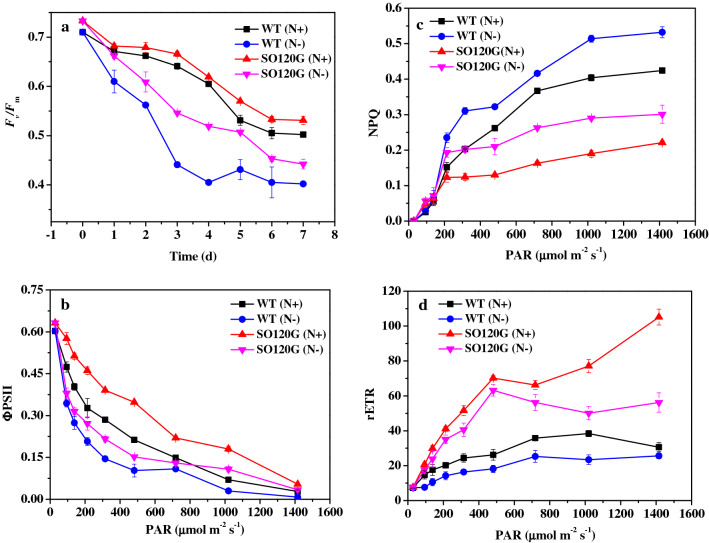
Chlorophyll fluorescence parameters of the WT and SO120G under the N+ and N− culture conditions. (**a**) The *F*_*v*_/*F*_*m*_ values of the WT and SO120G; (**b**) the ΦPSII values of the WT and SO120G; (**c**) the NPQ values of the WT and SO120G; and (**d**) the rETR of the WT and SO120G.

The mutant SO120G showed a higher lipid productivity than that of the WT. To elucidate the mechanism responsible for the enhanced lipid biosynthesis metabolism of SO120G under nitrogen limitation conditions, the photosynthetic efficiency, including ФPSII, NPQ, and rETR, of the SO120G and WT was determined. The value of *F*_*v*_/*F*_*m*_ reflected the potential maximum quantum efficiency of the PSII. The *F*_*v*_/*F*_*m*_ value of SO120G was consistently greater than that of the WT, even when cultivated under stress (N-) conditions (Fig. 3a). The results of Fig. 3b show that the variation tendency of the ФPSII values of the SO120G and WT decreased with gradually increasing photosynthetically active radiation (PAR, 29–1416 μmol photons·m^−2^ s^−1^). ФPSII indicates the actual photosynthetic efficiency of PSII, and the ФPSII values of SO120G were consistently higher than that of the WT over the entire investigated light step ranges (*p* < 0.05). All of the above results preliminary indicated that the mutant SO120G might have the potential to utilize light energy much more effectively than the WT during the lipid accumulation phase, which might result in more efficient biomass and lipid productivity.

The dynamic characteristics of non-photochemical quenching (NPQ) were observed and analyzed during photosynthetic induction. The NPQ value typically has been used to reflect a photo protection process where excess light energy that is not being utilized in photosynthesis will be dissipated due to thermal radiation^13,28^. As shown in Fig. 3c, the NPQ values of the SO120G and WT strains increased with increasing PAR. The NPQ values of SO120G were lower than that of the WT strain over an illumination intensity ranging from 139 to 1416 μmol photons·m^−2^ s^−1^ (*p* < 0.05), suggesting that SO120G exhibited less thermal dissipation than the WT strain at a high irradiance. The rETR, known as the relative electron transport rate, is considered to be an approximation rate of electrons pumped through a photosynthetic chain^13,29^. As shown in Fig. 3d, the rETR of the SO120G and WT strains changed in response to the increasing PAR and reached a maximum peak at 1416 and 721 μmol photons·m^-2^·s^−1^ respectively. The rETR values of the mutant SO120G were higher than that of the WT strain over an irradiance range 29–1416 μmol photons·m^−2^ s^−1^ (*p* < 0.05), indicating that the SO120G obtained higher photosynthetic electron transport rates than the WT strain. In summary, the mutant SO120G showed a higher actual photosynthetic efficiency and an efficient electron transport rate. In addition, SO120G showed a lower thermal dissipation ability and stronger resistance to high PAR than the WT strain. The chlorophyll *a* and carotenoid contents in the WT and mutant SO120G cells were quantitatively analyzed (Table 2). Furthermore, the nitrogen conditions had a more significant effect on pigment accumulation in the mutant compared with the WT. Under the N+ conditions, the pigment contents were higher in SO120G than the WT. In addition, the Chl *a* content decreased under the N− culture condition, but the carotenoid content of the WT and SO120G increased noticeably under the N− conditions.

The improved photosynthetic efficiency of SO120G may attribute to the increased expression of the peripheral antenna complexes associated with the PSII, which further enhanced the electron transport efficiency of the respiratory chain between the PSI and PSII and led to an increase in photosynthetic pigment, including the Chl *a* and carotenoids content, and a higher biomass in the SO120G than in the WT even when cultivated under further stress (N−) conditions. In photosynthesis, photosynthetic pigment not only has light-harvesting complexes antenna proteins but also has the reaction centers of PSII and PS1^13^, and Chl *a* and carotenoids are the primary photosynthetic pigments in the chloroplasts and indicators of photosynthetic efficiency in the microalgae. In addition, it has been proven that a decrease in starch synthesis will cause repartitions of energy in the PSII in *Nicotina sylvestris*^30^. The blocked competitive starch synthesis pathway may facilitate carbon flux repartitioning into lipid synthetic metabolism, but it may also result in a decrease in the photosynthetic efficiency and biomass. These results suggest that the mutant SO120G exhibited a higher photosynthetic efficiency under nutritional stress, which will contribute to much more photosynthetically fixed carbon redirected into lipid biosynthesis pathways.

### Overview of transcriptome sequencing and annotation of *S. obliqnus*

#### Influence of ^12^C^6+^ Ion irradiation on transcriptome sequencing

The *S. obliqnus* mutant SO120G with enhanced photosynthesis efficiency and lipid productivity was screened. To further explore the metabolic response induced by ^12^C^6+^ ion irradiation, the transcriptomic profiles of SO120G and the WT were investigated. The mutant SO120G (named as SO120G-1, SO120G-2, and SO120G-3) and the wild type were considered as the controls (named as SOWT-1, SOWT-2, and SOWT-3). Assessing the relevance of biological replicates is important for analyzing transcriptome sequencing data.

**Figure 4:**
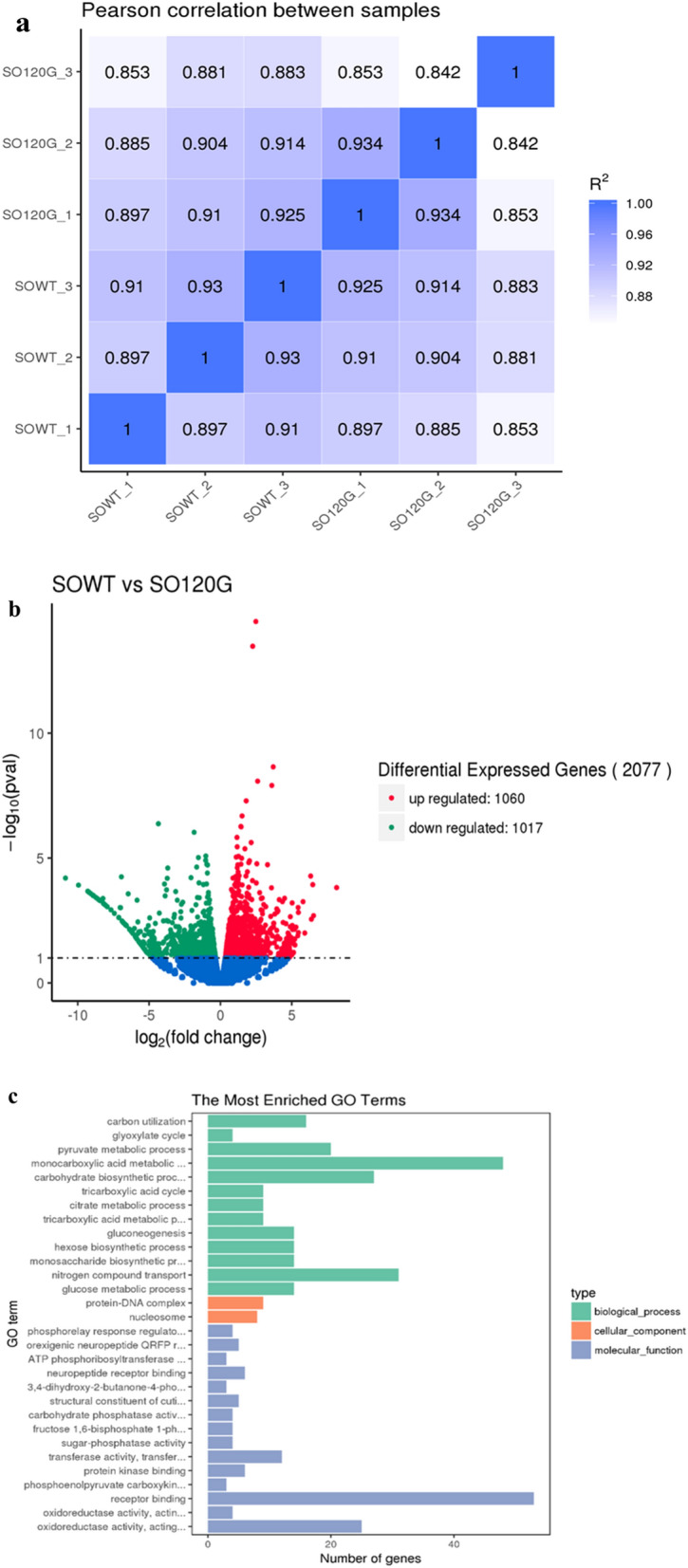
(**a**) Correlation plot between the different samples. The control group (SOWT-1, SOWT-2, and SOWT-3), experimental group (SO120G-1, SO120G-2, and SO120G-3); (**b**) the MA plot of the differentially expressed genes (DE genes). Significantly up-regulated and down-regulated genes are shown as red and green dots, respectively. Genes with no significant changes are shown as black spots; (**c**) gene ontology (GO) classification analysis of all of the genes and DE genes. The X-axis shows the number of genes, the left Y-axis shows the GO function.

The correlation coefficient results between the six samples are shown in Fig. 4a. The samples within the same treatment condition showed a higher Pearson correlation, while a lower correlation was observed between the WT and SO120G. The result indicates that the transcriptome data were reliable. A total of 2077 genes with at least a twofold change were identified as differentially expressed genes (DEGs), of which 1060 genes were up-regulated, and 1017 genes were down-regulated (Fig. 4b).

Furthermore, a gene ontology (GO) classification was performed according to the gene annotations. All of the DEGs were distributed among the three GO categories: biological process, cellular component, and molecular function (Fig. 4c). The results showed that the most active biological processes were “monocarboxylic acid metabolic” and “nitrogen compound transport”. The primary molecular functions were “receptor binding” and “oxidoreductase activity”. Moreover, genes involved in the biological processes of carbohydrate biosynthetic processes were significantly downregulated in the SO120G, which confirms the increased growth results shown in Fig. 2a.

#### Influence of ^12^C^6+^ ion irradiation on S. obliqnus cellular metabolism

The transcript abundance of the fatty acid, pigment, photosynthesis, and the starch and protein biosynthesis pathways were analyzed. Most of the DEGs involved in fatty acid biosynthesis were significantly up-regulated in the mutant SO120G group. Among these genes, 3-oxoacyl-[acyl-carrier protein] reductase (FabG), acyl-[acyl-carrier-protein] desaturase (FAB2, SSI2, DESA1), long-chain acyl-CoA synthetase (ACSL), and fatty acid desaturase (FAD) were associated with fatty acid biosynthesis, and very-long-chain (3R)-3-hydroxyacyl-CoA dehydratase (HACD, PHS1, PAS2) and 3-ketoacyl-CoA synthase (KCS) were related to the termination of fatty acid chain elongation, and these four genes were involved in very long-chain fatty acid synthesis corresponding to C20:5 and C22:6 in Table 3. In addition, most of the DEGs involved in unsaturated fatty acids biosynthesis were significantly up-regulated in the mutant SO120G group, including the FabG, PHS1, PAS2, DESA1, and Acyl-CoA Oxidase (ACOX1, ACOX3). The above genes were also associated with oleic acid production, which is a desirable biodiesel material. Most importantly, it was found that the key gene, FabG, catalyzing malonyl-ACP generation from malonyl-CoA and FabD, being responsible for the de novo biosynthesis of fatty acids, were highly overexpressed in the mutant SO120G group. These DEGs involved in fatty acid biosynthesis were also reported in *Dunaliella. bardawil* under heat stress^31^ and in *Chromochloris zofingiensis* in nitrogen deprivation conditions^32^. The significant overexpression of these genes suggests that the synthesis of fatty acids was enhanced in the mutant SO120G group.

**Table 3:** Fatty acid profiles of lipids derived from the WT and SO120G samples under N+ and N− cultivations.

Fatty acids	Fatty acid content (%)			
	WT (N+)	WT (N−)	SO120G (N+)	SO120G (N−)
C14:0	0.44 (± 0.02)	0.21 (± 0.02)	0.46 (± 0.02)	0.11 (± 0.02)
C16:0	21.18 (± 0.42)	10.04 (± 0.42)	20.78 (± 1.02)	18.21 (± 0.62)
C16:3	5.16 (± 0.54)	16.31 (± 0.82)	8.11 (± 0.32)	12.54 (± 0.02)
C18:0	5.19 (± 0.62)	2.32 (± 0.04)	6.17 (± 0.12)	4.32 (± 0.02)
C18:1	12.34 (± 0.21)	15.32 (± 0.29)	45.13 (± 5.02)	43.33 (± 2.02)
C18:3	35.26 (± 0.64)	36.32 (± 1.32)	8.31 (± 0.03)	13.21 (± 0.04)
C20:5	2.39 (± 0.25)	0.33 (± 0.01)	4.48 (± 0.05)	2.83 (± 0.01)
C22:6	18.04 (± 2.21)	19.15 (± 1.32)	6.57 (± 0.02)	5.45 (± 0.02)
SFA	26.81 (± 1.02)	12.57 (± 0.62)	27.41 (± 1.32)	22.64 (± 0.62)
MUFA	12.34 (± 0.25)	15.32 (± 0.54)	45.13 (± 6.12)	43.33 (± 1.32)
PUFA	60.85 (± 5.02)	72.11 (± 4.32)	27.47 (± 1.12)	34.03 (± 2.43)

Values are mean (± SD) of n = 3 cultivations per treatment.

**Figure 5:**
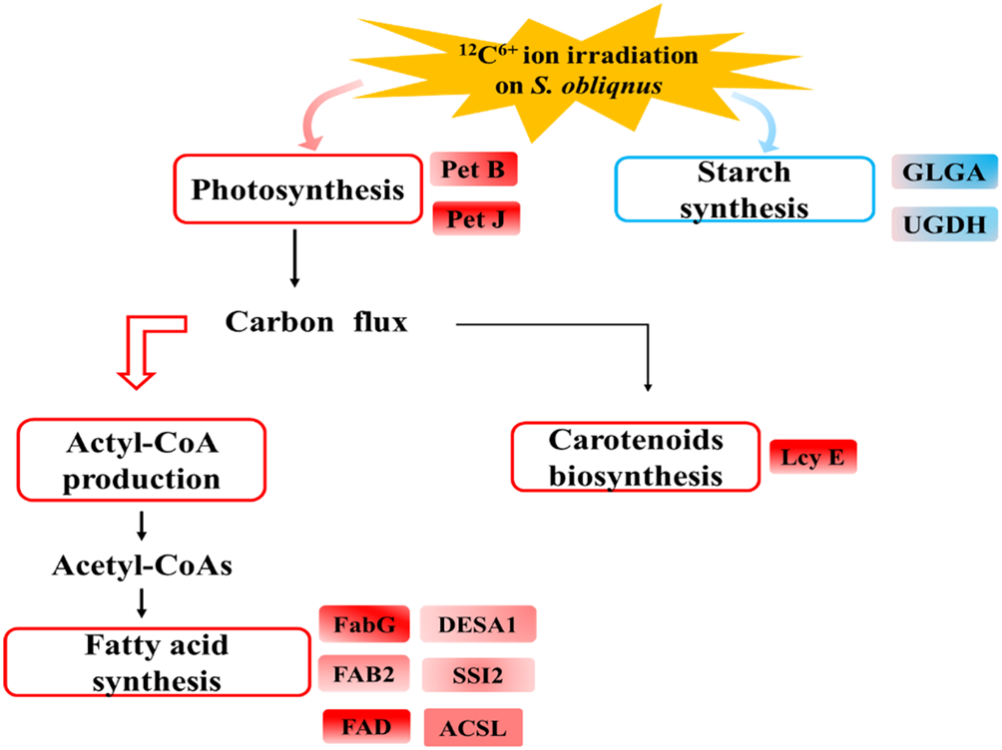
A mechanistic model for ^12^C^6+^ ion irradiation on *S. obliqnus*. FabG, 3-oxoacyl-[acyl-carrier protein] reductase; FAB2, SSI2, DESA1, acyl-[acyl-carrier-protein] desaturase; ACSL, long-chain acyl-CoA synthetase; FAD, fatty acid desaturase; Pet B, cytochrome b6; Pet J, cytochrome c6; UGDH, UDPglucose 6-dehydrogenase; GLGA, starch synthase; Lcy E, lycopene epsilon-cyclase. The boxes in red and blue indicate the up- and down-regulated pathways, respectively. Significantly up-regulated and down-regulated genes are shown as the red and blue rectangles.

Since the fatty acid contents increased with photosynthesis activity and increased during cultivation of *D. salina* (Figs. 2, 3) in the mutant SO120G, the genes involved in the photosynthesis and β-carotene biosynthesis were analyzed in the SO120G. The key gene cytochrome, b6 (Pet B), for the cytochrome b6/f complex and the cytochrome, c6 (Pet J), for photosynthetic electron transport were significantly up-regulated in SO120G. The enzymes involved in starch and pigment metabolism were also examined. The results showed that the synthesis of starch (UDPglucose 6-dehydrogenase (UGDH) and starch synthase (glgA)) were down-regulated in the mutant SO120G group. Additonally, the key gene, lycopene epsilon-cyclase (Lcy E), which is involved in the biosynthesis of the carotenoids pathway was up-regulated in the SO120G. The transcriptome data showed that the synthesis of energy storage molecules (starch) decreased, but the level of photosynthesis (pet J and pet B) increased due to increased carotenoid metabolism (LcyE) in the mutant SO120G (Fig. 5). This finding can be ascribed to the reallocation of cellular carbon and ATP from biological functions, such as photosynthesis, to the synthesis of energy storage molecules under stress conditions^19^.

## Conclusions

*S. obliqnus* was mutated using ^12^C^6+^ irradiation from heavy carbon ions, and a mutant strain SO120G with a 57% increment in biomass yield was obtained. The photosynthetic efficiency of the cells was analyzed to elucidate the improved lipid parameters. SO120G exhibited a 2.4 fold higher in lipid yield than the WT when they were cultivated in the nitrogen-limited conditions, likely owing to an improved quantum efficiency of photosynthesis under stress conditions. Moreover, the transcriptome data indicated that most of the DEGs involved in the biosynthesis of fatty acids were significantly up-regulated, and those involved in photosynthetic activity were up-regulated in the SO120G. Overall, ^12^C^6+^ irradiation induced fatty acids caused by changes in the photosynthetic efficiency and energy metabolism in the mutant SO120G cells.

## Data Availability

RNA-seq reads for one *S. obliqnus* WT sample and two *S. obliqnus* mutant SO120G samples are available at the National Center for Biotechnology Information SRA database under the accession PRJNA805302. The RNA-seq reads of the other reported samples are not available due to file corruption during storage.
