# Author Correction: Characterization and RNA-seq transcriptomic analysis of a *Scenedesmus obliqnus* mutant with enhanced photosynthesis efficiency and lipid productivity

**DOI:** 10.1038/s41598-022-09012-3

**Published:** 2022-03-21

**Authors:** Yimei Xi, Liang Yin, Zhan you Chi, Guanghong Luo

**Affiliations:** 1 Gansu Engineering Technology Research Center for Microalgae, Hexi University, Zhangye, 734000 People’s Republic of China; 2 School of Bioengineering, Dalian University of Technology, Dalian, 116024 People’s Republic of China

Correction to: *Scientific Reports* https://doi.org/10.1038/s41598-021-88954-6, published online 03 June 2021

The original version of this Article contained an error.

The Data Availability section was omitted. It has now been included and states:

**Data Availability**

RNA-seq reads for one *S. obliqnus* WT sample and two *S. obliqnus* mutant SO120G samples are available at the National Center for Biotechnology Information SRA database under the accession PRJNA805302. The RNA-seq reads of the other reported samples are not available due to file corruption during storage.

The original Article has been corrected.

